# Formative evaluation of a telemedicine model for delivering clinical neurophysiology services part II: The referring clinician and patient perspective

**DOI:** 10.1186/1472-6947-10-49

**Published:** 2010-09-15

**Authors:** Patricia Breen, Kevin Murphy, Geraldine Browne, Fiona Molloy, Valerie Reid, Colin Doherty, Norman Delanty, Sean Connolly, Mary Fitzsimons

**Affiliations:** 1 Epilepsy Programme, Beaumont Hospital, Dublin 9, Ireland; 2 Department of Neurology, Sligo General Hospital, Sligo, Ireland; 3 Department of Clinical Neurophysiology, Beaumont Hospital, Dublin 9, Ireland; 4 Department of Neurology, St. James's Hospital, James's Street, Dublin 8, Ireland; 5 Department of Clinical Neurophysiology, St. Vincent's University Hospital, Elm Park, Dublin 4, Ireland

## Abstract

**Background:**

Feedback from service users will provide insight into opportunities for improvement so that performance can be optimised. In the context of a formative evaluation referring clinician and patient satisfaction with a teleneurophysiology service was examined during a 20 week pilot period.

**Methods:**

Questionnaire surveys of referring clinicians and patients were conducted.

**Results:**

Fifteen (58%) clinicians responded to the first part of a postal survey which examined their satisfaction with traditional clinical neurophysiology services. Nine (35%) responded to a second part which assessed their experience with the teleneurophysiology service. Teleneurophysiology improved satisfaction with waiting times, availability of results and impact on patient management. There was unanimous support from the clinicians for the permanent development of a teleneurophysiology service, although 2 cautioned this could delay establishing a neurology service in their region.

Eighty-two percent (116/142) of patients responded to a survey of their satisfaction with teleneurophysiology. This was compared to a previous report of 322 patients' experience with traditional CN services in Ireland. Waiting times for appointment were shorter for the former group who supported the telemedicine model recognising that it reduced the travel burden and need for overnight journeys. The two groups were equally anxious about the investigation although the teleneurophysiology patients received more prior information.

**Conclusion:**

This study illustrates that teleneurophysiology is an acceptable model of service delivery for its primary customers. Their feedback is important in informing appropriate design and governance of such innovative models of health service provision.

## Background

Clinical neurophysiology (CN) involves the recording and assessment of bioelectric signals originating in the nervous system to evaluate its integrity. Traditional CN requires the patient to travel to a specialist central department to have these bioelectric signals recorded. With teleneurophysiology, data acquisition takes place at a satellite centre from where the data is communicated to a central department for analysis [1]. To rephrase Jarvis and Stanberry [2], teleneurophysiology is the point-to-point communication of bioelectric signal data from one location to another for the purpose of interpretation or consultation. Fundamentally, the data rather than the patient does the travelling. Teleneurophysiology can improve access to CN services and expert opinion for patients and referring clinicians at remote under-served sites [3,4]. In the context of burgeoning demand this model of service delivery has the potential to refocus limited resources and increase efficiencies [5,6].

To test this potential, a teleneurophysiology service providing routine EEG investigation was established and assessed [5]. A formative evaluation was conducted to examine the teleneurophysiology model in terms of its utility, technical performance and stakeholder satisfaction (clinical neurophysiology service providers, patients and referring clinicians). Formative or constructive evaluation is conducted the early stages of an implementation to assess its operational aspects and identify any changes to the service that are required to stabilise or optimise performance. This study reports on the teleneurophysiology service performance from the perspective of the patient and referring clinician. Technical and health service provider aspects of the evaluation are reported in an accompanying paper[5].

The primary customers of neurophysiology services are patients and referring clinicians. The referring clinician requires the expert CN opinion to guide the management of their patient. This requirement makes them, as well as their patients, users of the service. As with any newly introduced service, the consumer feedback is an essential component of its formative evaluation. Satisfaction surveys help assess performance of a service, further the understanding of customer needs and expectations, and expose opportunities for improvement. They are considered an important component of quality assurance programmes by bringing customer preferences into the quality assessment process [7-9]. The purpose of this study was to survey the satisfaction of referring clinicians and patients with the teleneurophsyiology model.

## Methods

Over a twenty week period, during which 40 clinical sessions were conducted, a teleneurophysiology service which provided an EEG service was piloted and evaluated. A quasi-experimental one-group pretest-posttest design was employed to assess customer satisfaction with the teleneurophysiology model [10,11]. The medical research ethics committees of both Beaumont Hospital and Sligo General Hospital reviewed and approved the study.

### Context of the study

The context of this study is fully described a related paper [5]. Beaumont Hospital on the east coast of Ireland provided the host expert CN department while the satellite centre was set up 130 miles away in the northwest region at Sligo General Hospital (SGH). Prior to the pilot only traditional CN services, based at either Dublin or Galway (85 miles from SGH), were available to the clinicians and patients of the northwest of Ireland. The consequent geographical inequities of this have previously been described [4].

### Study participants

The study participants included medical consultants in practice in the northwest of Ireland who referred patients to the teleneurophysiology service at SGH. Referred patients who attended for EEG recording at SGH were also invited to take part.

### Data collection

#### Referring clinical satisfaction

Referring clinician satisfaction data was collected by means of a detailed postal questionnaire delivered in two parts. Part one of the of the questionnaire was administered to collect pre-teleneurophysiology intervention observations while part two aimed to assess post-intervention observations. In this regard, the clinician satisfaction survey employed a pretest/posttest study design.

On receipt of a referral to the teleneurophysiology service, part one of the questionnaire together with information and consent form was sent by post to the referring clinician. Therefore, part one was essentially a survey of satisfaction with traditional clinical neurophysiology services and included questions on the profile of their clinical practice, their requirements for clinical neurophysiology, their perception of the impact of clinical neurophysiology investigation on patient management, and their perception of traditional clinical neurophysiology services in Ireland.

Part two of the questionnaire was sent to referring clinicians at the end of the twenty week pilot phase. This second part questioned the clinicians on aspects of the teleneurophysiology service including its impact on patient management, service quality and their perspective on establishing a permanent teleneurophysiology service for the region. Both parts of the questionnaire included spaces for respondents to add any additional comments and they were invited to continue these on a separate sheet if necessary. Referring clinicians were provided with pre-addressed stamped envelopes to return the questionnaires.

#### Patient satisfaction

Patient satisfaction data was collected immediately after the patient's teleneurophysiology EEG recording at SGH. Patients, or their escort, were given information about the satisfaction survey and if happy to participate were asked to complete a consent form and a questionnaire before leaving the EEG centre. Questions regarding the teleneurophysiology appointment, waiting time for appointment, satisfaction with prior information about the EEG and arrangements made to get to the teleneurophysiology centre were asked. Patients were asked to add any additional comments and if necessary continue these on a separate sheet. It was intended that any such comments would provide further insight into the patients' experience. This data represents the posttest (after the teleneurophysiology intervention) observation. Pretest observation data was provided from a previously reported study of patient satisfaction with traditional CN services in Ireland [12].

### Data analysis

Data from the satisfaction questionnaires were tabulated and from this totals and proportions or percentages in different categories were established. Pearson's chi-square test was used to assess the difference in proportions between parts 1 and 2 (pre and post intervention) of the clinician survey.

Responses from the survey of patient satisfaction with the teleneurophysiology model (post-intervention observations) were compared with data from a previously reported survey of satisfaction with the traditional model of CN service delivery[12]. In that previous study 322 patients who attended 6 different CN centres in Dublin responded to a satisfaction questionnaire. These pre-intervention observations included data for both paediatric and adult patients, for all CN investigation modalities (EEG, EMG, NCS, and EP) and for patients resident in a region with and without a local CN service [5]. As the pre and post-intervention data were not equivalent, the pre-intervention data represent a control period rather than a control group and provided a baseline for a preliminary comparison of the two models of service delivery. Pearson's chi-square test was used to compare proportions between the pre and post-intervention data.

Additional comments made by survey participants were also reviewed to further reveal service users, patients and their referring clinicians, perception of the teleneurophysiology model.

## Results

Over the 20 week teleneurophysiology pilot period, 40 separate clinic sessions were conducted during which 142 patients (74 female, 68 male) had an EEG investigation at SGH. Further details of the utilisation of the teleneurophysiology service are documented in an accompanying paper [5].

### Referring clinician

Twenty-six different consultant clinicians practicing in the northwest region of Ireland referred patients to the teleneurophysiology service during the pilot phase. Of these 15 (58%) responded to part one of the survey while 9 (35%) responded to part two. Not all survey questions were answered by each of the respondents. The range of different medical specialties that availed of the teleneurophysiology service is illustrated in the accompanying paper [5]. Tables 1 and 2 summarise responses to parts one and two of the survey respectively.

**Table 1:** Referring clinician satisfaction with conventional clinical neurophysiology

*Referring Clinician Survey Part I* SATISFACTION WITH CONVENTIONAL CN				*Total*
**Which aspect of clinical neurophysiology testing would you be more likely to avail of?**				
	
***EEG***	***EMG/NCS***	***EP***		
9	5	0		*14*

**Where are your patients referred to for CN investigation?**				
	
***Dublin***	***Galway***	***Dublin/Galway***	***Other***	
5	3	4	0	*12*

**What is the average waiting time for CN testing experienced by your patients?**				
	
***< 1 wk***	***1 wk - 1 mth***	***1 - 3 mth***	***> 3 mth***	
0	0	6	7	*13*

**What proportion of previous referrals were helpful in the management of patients?**				
	
***< 25%***	***25% - 50%***	***50% - 75%***	***> 75%***	
1	2	4	5	*12*

**Are CN reports readily available at the patient's follow-up appointment with you?**				
	
***Yes***	***No***			
4	7			*11*

**Does lack of a local service influence your decision to refer patients for CN investigation?**				
	
***Never***	***Sometimes***	***Always***		
1	10	2		*13*

**Because there is no local service, do you refer patients to other specialist consultants in preference to sending them for CN investigation?**				
	
***Yes***	***No***			
8	4			*12*

**Do you routinely send patients for an MRI/CT before referring them for an EEG?**				
	
***Yes***	***No***			
8	5			*13*

**Does the lack of a local CN service impact negatively on patient management?**				
	
***Yes***	***No***			
11	0			*11*

**Table 2:** Referring clinician satisfaction with teleneurophysiology

*Referring Clinician Survey Part II* SATISFACTION WITH TELENEUROPHYSIOLOGY				*Total*
**What is the average waiting time for CN testing experienced by your patients?**				
	
*< 1 wk*	*1 wk - 1 mth*	*1 - 3 mth*	*> 3 mth*	
*0*	*7*	*2*	*0*	*9*

**What proportion of teleneurophysiology referrals were helpful in the management of patients?**				
	
*< 25%*	*25%-50%*	*50%-75%*	*> 75%*	
*0*	*0*	*5*	*3*	*8*

**Are teleneurophysiology reports readily available at the patient's follow-up appointment with you?**				
	
*Yes*	*No*			*9*
*9*	*0*			

**Would you welcome the introduction of a permanent teleneurophysiology service in your region?**				
	
*Yes*	*No*			
*9*	*0*			*9*

**Do you feel there would be any negative implications with the introduction of a permanent teleneurophysiology service?**				
	
*Yes*	*No*			
*2*	*6*			*8*

In part one of the survey, 100% (12/12) of the responding clinicians considered that CN was relevant to their medical practice with 64% (9/14) indicating that they were more likely to avail of EEG services than other neurophysiology investigation modalities. Prior to the availability of the teleneurophysiology service clinicians referred their patients to either Dublin (130 miles from Sligo) or Galway (85 miles from Sligo) for investigation. All respondents (13/13) perceived that waiting time for traditional CN appointments was greater than 1 month with seven of these (54%) suggesting that patients wait longer than 3 months to be seen. All respondents agreed that the lack of a local service impacted negatively on patient management and indicated that this deficit either sometimes (77%) or always (15%) influenced their decision to refer patients for CN. Similarly, in the absence of a local service 67% (8/12) respondents reported that they would refer patients to other clinical specialities in preference to sending them for CN with 62% (8/13) indicating that they would routinely send patients for MRI or CT before referring for EEG. Seventy-five percent (9/12) of the referring clinicians believe that more than 50% of previous referrals were helpful in patient management, although 64% (7/11) said neurophysiology reports were often not available at the patient's follow-up appointment (table 1). Comments provided by respondents help to further contextualise these results (Additional file 1).

The second part of the survey documented the referring clinicians experience with the teleneurophysiology pilot service (table 2). Compared to the traditional services: the perceived waiting time for appointment was significantly reduced as 78% (7/9) reported that their patients received a teleneurophysiology appointment within 1 month of referral (p < .001); the proportion of respondents who believed that more than 50% of investigations were helpful in patient management rose significantly (p < .05) from 75% to 100% (8/8); and EEG reports were more promptly (p < .01) available (from 36% to 100%). One hundred percent (9/9) of the referring clinicians would welcome a permanent teleneurophysiology service in the northwest region. However, 25% (2/8) indicated that there may be negative implications with the introduction of such a service. Annotations included by respondents indicated that they had concerns that the establishment of a permanent service might delay the appointment of a consultant neurologist to the region. Additional comments provided by the referring clinicians further elucidate these responses (Additional file 2).

### Patient

Eighty-two percent (116) of the 142 patients who had an EEG investigation at the SGH teleneurophysiology centre returned completed questionnaires. Not all survey questions were answered by each of the respondents (table 3). Twenty-five percent (24/97) of the teleneurophysiology patients who responded reported having had a previous EEG investigation. Information about where those previous EEGs were conducted was provided by 16 respondents. Of these 14 had travelled to Dublin, 1 had gone to Galway and 1 had gone to both Galway and Dublin on separate occasions. Forty-seven percent (37/78) said that they took time off either school, college or work to attend the teleneurophysiology centre.

**Table 3:** Patient satisfaction with teleneurophysiology

*Patient Survey* SATISFACTION WITH TELENEUROPHYSIOLOGY				*Total*
**Is this the first time you have had an EEG?**				
	
*Yes*	*No*			
*73*	*24*			*97*

**How long did you wait from the time your appointment was made to the date of your test?**				
	
*< 1 wk*	*1 wk - 1 mth*	*1 - 3 mth*	*> 3 mth*	
*11*	*62*	*16*	*2*	*91*

**Did you have to take time off school/college/work for your appointment?**				
	
*Yes*	*No*			
*37*	*41*			*78*

**Did someone accompany you to your appointment?**				
	
*Yes*	*No*			
*67*	*15*			*82*

**Who accompanied you?**				
	
*Family member*	*Friend*	*Other*		
*49*	*3*	*15*		*67*

**Did he/she take time off school/work to accompany you?**				
	
*Yes*	*No*			
*33*	*30*			*63*

**Was the reason for the test explained to you by your doctor?**				
	
*Yes*	*No*			
*76*	*16*			*92*

**Were you anxious about the test?**				
	
*Yes*	*No*			
*46*	*47*			*93*

**Do you have an appointment to return to the doctor who sent you for this test?**				
	
*Yes*	*No*			
*38*	*34*			*72*

A previous examination of traditional CN services in Ireland reported the satisfaction of 322 patients and provides a baseline for interpreting this patient survey [12]. The teleneurophysiology pilot resulted in 80% (73/91) of patients being seen within 1 month of referral whereas 50% (157/313) of patients were seen within this time frame with traditional CN services. Similar percentages of patients attending traditional and teleneurophysiology services reported that the reason for investigation had been explained to them by their doctor (80% - 241/305 and 83% - 76/92 respectively). Likewise, equal proportions of patients attending either model of service delivery reported being anxious about the test (43% - 130/305 in traditional service and 46% - 76/92 in teleneurophysiology service). However, a notably larger proportion of teleneurophysiology patients (73% - 73/99 compared to 26% - 81/308) reported receiving written information about the test procedure in advance and knowing how long the test would take (86% - 83/97 against 35% - 109/308). Compared to traditional CN services more patients were accompanied to the teleneurophysiology centre (82% - 67/82 versus 65% - 184/285). In 52% (33/63) of the teleneurophysiology cases, the accompanying person also took time of either school or work to escort the patient. Thirty-eight (53%) respondents said that they had an appointment to return to the doctor who sent them to the teleneurophysiology centre compared to 70% (191/273) in the traditional CN service study. While 4% (12/292) of attendances at traditional CN services have been reported to necessitate an overnight journey, none of the teleneurophysiology EEG appointments required this. Comments provided by respondents further describe the patients' perspective on the teleneurophysiology service (Additional file 3).

## Discussion

When introducing a new or changed service it is appropriate to understand the needs, and expectations of the people who will use the service [13]. This study illustrates both patient and referring clinician readiness for a telemedicine model of CN service delivery. Feedback from patients was positive indicating that teleneurophysiology helps meet their needs with minimal travel involved and a reduced waiting time for the one-hour test. Referring clinicians consider teleneurophysiology to be beneficial in the clinical management of patients with improved access to an informative test. This user acceptance of the model together with improved access to service and its cost effectiveness, reported in an accompanying manuscript [5], demonstrate potential for improved quality, safety and efficiency with the introduction of teleneurophysiology.

Part one of the referring clinician survey illustrated a dissatisfaction with the availability of traditional CN services and highlighted opportunities for improvement by the introduction of a teleneurophysiology service. The indication was that they are discouraged from referring patients as delays in getting an appointment can often make referral irrelevant in terms of managing the patient's condition. Where tests are carried out there are often delays in receiving the report. In addition, patients may be reluctant to travel for investigation. These findings echo those of a previously reported survey of referring clinician needs, expectations and satisfaction with CN services in Ireland [12]. The second part of the survey showed that teleneurophysiology improved referring clinician satisfaction in terms of waiting time and impact on patient management. There was absolute support for continuing the teleneurophysiology service.

The main observations from the patient survey were that teleneurophysiology can reduce geographical inequities by extending CN services to under-served sites. The proportions of teleneurophysiology and traditional CN patients who reported being anxious about the investigation were similar despite the former group receiving more prior information. Teleneurophysiology eased the travel burden on patients and their families and eliminated a need for overnight accommodation to facilitate CN investigation. It also resulted in reduced waiting times for appointment for EEG investigation compared to traditional CN.

Study motivation is the main difference between this and previously reported evaluations of teleneurophysiology [14-16]. The focus of evaluation of a health informatics system will depend on where it is in its life-cycle [17]. Identification of potential solutions motivates the exploration phase while technical feasibility and user acceptance of a particular solution is assessed during technical development phase. During the adaptation phase the system is evaluated within controlled conditions to examine how well it works in practice and to identify any adjustments required to optimise its performance. In the expansion phase the optimised system is extended to more users and more applications, and summative or outcome evaluation becomes the focus [17]. Previously reported teleneurophysiology studies were motivated by demonstrating technical feasibility and user acceptance [14-16]. By comparison, this study was an adaptation phase evaluation to determine which aspects of the teleneurophysiology system work well and which parts may need improvement. In this regard the concept of system covers technical structure and process features.

In Ireland the need for more clinical neuroscience resources has been acknowledged [18]. Teleneurophysiology can enhance efficiency and effectiveness of limited resources. It is technically achievable, acceptable to service providers [5,14-16], referring clinicians and patients and the unit cost per investigation is comparable in both the traditional and teleneurophysiology mode of service delivery [5]. It is well suited to the delivery of a routine EEG service, although some of the more complex CN procedures may still require patients to attend in person at a traditional centre. With teleneurophysiology, patients, clinicians and healthcare managers benefit from having local, fast access to important investigations for people with neurological symptoms. Although, the tele-based service has comparable costs to a traditional CN department, the advantage to the patients including: more patients receive an expert neurophysiology opinion; earlier diagnosis; early more rational and safer treatment; provision of consistent CN services to geographically diverse areas; and reduction in waiting lists [19,20], as well as more equitable access to services from a referring clinician perspective are the arguments for implementing the described teleneurophysiology service.

## Limitations

As previously acknowledged, this study does not prove causality between the telemedicine model and customer satisfaction [5]. For example, the pre and post-intervention patient groups in the patient satisfaction survey were not equivalent in a number of respects including age profile, CN investigation modality referred for, CN centre attended and region of residence (e.g. availability of local CN). Furthermore, due to unmet demand [4], waiting times for traditional CN services were considerably longer than for the teleneurophysiology service. The reduced waiting time may have positively biased the satisfaction of the post-intervention group. Without controlling these variables it cannot be claimed that the teleneurophysiology model is solely responsible for the level of patient satisfaction.

Twenty-five percent (24/97) of the teleneurophysiology patients had previously had an EEG at another centre. It may have been possible for this group to act as their own controls by asking them additional questions about their prior EEG investigation experience. These were not included as it was not known in advance the proportion of patients that were likely to have had a previous EEG. Also, the time span between their EEG investigations may have biased results. Another possible limitation of the patient questionnaire was the lack of questions regarding satisfaction with their clinician. However, the patient questionnaire was designed to specifically examine patient acceptance of the teleneurophysiology model.

The low response rate to parts one and two of the referring clinician questionnaire together with incomplete item response indicates self-selection and may have caused bias in the survey response. For example, those who chose not to participate may have been either more or less satisfied with the traditional CN and teleneurophysiology service models. Therefore, reasons other than the teleneurophysiology service may have influenced the findings of the clinician survey.

The pre-intervention measurements in the surveys represent data from a control period rather than data from a control group [10]. In this regard the study contributes to a formative evaluation that can inform further development of a teleneurophysiology service. Definitive verification of the model and its sustainability will require prospective, randomised controlled studies.

## Conclusion

In a globalised world international teleneurophysiology, where recordings made in one country are reviewed and interpreted by overseas clinicians, is a realistic concept [2,6]. Where shortages in health service resources exist, there may be opportunities to outsource for services from other countries. As the model crosses over traditional organisational boundaries and potentially international borders, professional, clinical, ethical and legal implications of teleneurophysiology need to be fully understood so that appropriate governance of such services can be put in place [2,21]. Essential to this is engagement with stakeholders, including service users, whose feedback will inform the delivery of a safe, efficient and acceptable service.

## Competing interests

The authors declare that they have no competing interests.

## Authors' contributions

PB, KM, GB, FM and MF were involved in the conception and design of the study, the collection of data, its analysis and interpretation. The process of the study and interpretation of data were further reviewed and monitored in detailed discussion with VR, CD, ND and SC. The manuscript was prepared for publication by PB and MF with critical review from the remaining authors. All authors approved the final version.

## Pre-publication history

The pre-publication history for this paper can be accessed here:

http://www.biomedcentral.com/1472-6947/10/49/prepub

## Supplementary Material

Additional file 1 **Additional comments provided by respondents to part one of referring clinician survey - satisfaction with conventional clinical neurophysiology**. These comments help to further contextualise the referring clinicians' satisfaction with the conventional model of clinical neurophysiology service delivery. Click here for file

Additional file 2 **Additional comments provided by respondents to part two of referring clinician survey - satisfaction with teleneurophysiology**. These comments help to further elucidate the referring clinicians' responses to the survey of their satisfaction with the telemedicine model of clinical neurophysiology. Click here for file

Additional file 3 **Additional comments provided by patients or their carers to the patient survey - satisfaction with teleneurophysiology**. These **c**omments further describe the patients perspective on the teleneurophysiology service model. Click here for file
