# Supplementary material for: Formative evaluation of a telemedicine model for delivering clinical neurophysiology services part II: The referring clinician and patient perspective
Source: BMC Med Inform Decis Mak. 2010 Sep 15;10:49. doi: 10.1186/1472-6947-10-49 (PMC2946265; doi:10.1186/1472-6947-10-49)
Supplement: Additional file 1 — Additional comments provided by respondents to part one of referring clinician survey - satisfaction with conventional clinical neurophysiology. These comments help to further contextualise the referring clinicians' satisfaction with the conventional model of clinical neurophysiology service delivery. [file 1472-6947-10-49-S1.DOC]

*“ I must add that I do receive very good support from UCGH neurology consultants. Always available to advise by phone which is appreciated”*

*“ most of the patients referred have refused to go ahead with the test. Too far away, too long to wait. No specialist review post test. In conclusion, an appalling situation.”*

*“To do an EEG is often good practice as part of a full organic work up. The long wait for appointment often means management has commenced by the time the EEG done or diagnosis made in the absence of the EEG (i.e. many psychotic diagnoses or dementia). Given the high incidence of epilepsy in learning disabled population an EEG is considered good practice and I welcome the opportunity to have it done at Sligo General.”*

*“I am aware that the number of people referred from the North West for neurophysiology testing is small. This does not reflect the need but the absence of such a service ………and the unacceptable delay in getting appointments in Dublin.”*

*“EEG very useful for diagnosis of epilepsy but long waiting time.”*

*“ Should Donegal citizens have specialist care of their neurological problems? Any modern health system should provide this care.”*

*“Large percentage of my patients endure behavioural and mental health problems in addition to having epilepsy (and an intellectual disability). Has great implication is terms of diagnostic clarity, management particularly in terms of addition of psychotropic medication most of which are pro-convulsant.”*

*“ We need a neurologist with clinical neurophysiology. Patients are hugely disadvantage – we are following up specialist neurological conditions in general clinics. No paramedical infrastructure – patients do not want to travel (constantly refuse).”*

*“Generally EEG has not been done by time I see patient for next appointment.”*

*“Invariably these results are not available and we have to get results faxed to OPD for consultation.”*

*“After neurophysiology investigation…….request input from a neurologist which at present can be up to 2 years for an appointment for a public patient and 6 months for a private patient.”*

*“The absence of a neurologist in the North West speaks for itself. I do not have to list (nor would I have the time to) the disadvantages and dangers of this deficiency.”*

*“No neurology service at present. Appalling wait time for Galway.”*

**Additional comments provided by respondents to part one of referring clinician survey – satisfaction with conventional clinical neurophysiology.**
