# Supplementary material for: Formative evaluation of a telemedicine model for delivering clinical neurophysiology services part II: The referring clinician and patient perspective
Source: BMC Med Inform Decis Mak. 2010 Sep 15;10:49. doi: 10.1186/1472-6947-10-49 (PMC2946265; doi:10.1186/1472-6947-10-49)
Supplement: Additional file 2 — Additional comments provided by respondents to part two of referring clinician survey - satisfaction with teleneurophysiology. These comments help to further elucidate the referring clinicians' responses to the survey of their satisfaction with the telemedicine model of clinical neurophysiology. [file 1472-6947-10-49-S2.DOC]

*“ I would welcome this service remaining though to date I’ve referred few patients. EEG is useful in differentiating depressive disorders associated with cognitive symptoms from organic brain syndromes. It’s useful in evaluating atypical dementia presentations.”*

*“It can be difficult at times to interpret EEG reports – reports received to date from teleneurophysiology have been very clear and helpful.”*

*“a good readily accessible service with quick reports available.”*

*“ would prefer if neurologist on site in order to discuss cases.”*

*“Quality of service and care to neurological patients negatively affected by lack of locally based neurologist and clinical neurophysiology. Pilot project encouraging us to refer more patients to their benefit.”*

*“Excellent service. Feedback helped in relation to type of anti-epileptic medications.”*

*“Telephone feedback would have been nice with difficult cases. Recommendations regarding appropriate medications would have been gratefully appreciated.”*

**Additional comments provided by respondents to part two of referring clinician survey - satisfaction with teleneurophysiology.**
