# Supplementary material for: Formative evaluation of a telemedicine model for delivering clinical neurophysiology services part II: The referring clinician and patient perspective
Source: BMC Med Inform Decis Mak. 2010 Sep 15;10:49. doi: 10.1186/1472-6947-10-49 (PMC2946265; doi:10.1186/1472-6947-10-49)
Supplement: Additional file 3 — Additional comments provided by patients or their carers to the patient survey - satisfaction with teleneurophysiology. These comments further describe the patients perspective on the teleneurophysiology service model. [file 1472-6947-10-49-S3.DOC]

*“Having the service based in Sligo was very helpful in this case and negated the need for an overnight trip to Dublin. Minimised upset…..extremely beneficial when such examinations are called for.”*

*“Could this test be done at LGH (Letterkenny General Hospital) and information transmitted to Beaumont by the same means?”*

*“Excellent service, desperately needed to ensure meeting the needs of people with epilepsy and excellent providing a local service for clients with intellectual disability who would otherwise have to travel to Dublin.”*

*“It has taken over 2 years to get an appointment with a view to clarification of epileptic clearance.”*

*“My doctor … did not follow up with tests as promised at appointment so I had to organise and push for them myself which having gone private I feel is not satisfactory as it has held up another procedure for the last year which I require as they are awaiting these results.”*

*“Just like to say the service was great and everything went well.”*

*“Pilot study good idea. Save going all the way to Dublin for one hour test. Thanking you.”*

*“Had this looked into 4 years ago but dismissed as old age by specialist.”*

*“Before attending for test: I’d like to know what exactly happens my brain. Are currents/radiation passed through it? The information sheet implies 1. A recurrence of my original blackout could be induced by the test. 2. My hair may have to be cut or shaved to attach electrodes to my scalp. Neither happened!”*

*“It is absolutely fundamental that these services are offered in the North West of Ireland and in particular Sligo. Not alone is it difficult enough for patients anxiously awaiting to do test but the endurance of having to travel sometimes overnight to reach either Dublin or Galway is unfair and unjust.”*

*“I requested to change the time of my appointment as it was inconvenient. I was not accommodated. I understand that there can be unexpected delays, however the department does not appear to be ‘over-run’. It was 12.35 (appointment time 12.00) and I still have not been called. I will now be even more inconvenienced. I do not find this service to be efficient.”*

*“The test was very easy. It was not scareful (sic) and the technician was very good at answering all questions regarding EEG before test and after test. Thank you very much.”*

**Additional comments provided by patients or their carers to the patient survey – satisfaction with teleneurophysiology.**
